# Continuous-Variable Quantum Key Distribution Based on *N*-APSK Modulation over Seawater Channel

**DOI:** 10.3390/e27090990

**Published:** 2025-09-22

**Authors:** Lei Mao, Zhangtao Liang, Zhiyue Zuo, Hang Zhang, Yijun Wang

**Affiliations:** School of Automation, Central South University, Changsha 410083, China

**Keywords:** continuous-variable quantum key distribution, N-APSK modulation, seawater channel

## Abstract

A continuous-variable quantum key distribution (CVQKD) can be realized over the seawater channel, but the transmission of quantum signals in seawater media exhibits significant attenuation effects. Therefore, we propose an *N*-symbol amplitude and phase shift keying (*N*-APSK) modulation scheme to enhance the transmission performance of the CVQKD over the seawater channel. Specifically, an optimal *N*-APSK modulation scheme is designed based on the principle of maximizing the minimum Euclidean distance (MED). The simulation results show that the CVQKD protocol based on *N*-APSK modulation achieves a longer transmission distance over the seawater channel compared to the Gaussian modulation protocol. Additionally, increasing the value of *N* simultaneously expands the number of rings in the constellation diagram, further enhancing the communication distance. This study transfers modulation methods from the field of classical communications to the field of quantum communications, achieving a substantial improvement in communication distance and thereby promoting the integration of quantum communications and classical communications.

## 1. Introduction

A continuous-variable quantum key distribution (CVQKD) [1,2,3] is a branch of quantum communication in which Alice and Bob encode information using orthogonal components of modulated light fields and then establish a series of keys through a public channel. CVQKDs primarily consist of two types: fiber [4,5,6] and free-space [7,8,9]. Free-space communication has garnered increasing attention due to its flexible and practical system architecture. Currently, most free-space CVQKD research is based on atmospheric channels [10,11]. However, as quantum communication technology matures, the construction of a global CVQKD network has become an inevitable trend. In this process, the realization of underwater CVQKDs is an indispensable component. In Ref. [12], the feasibility of CVQKDs over the seawater channel was confirmed based on a Monte Carlo model. Ref. [13] analyzed the wavelength attack mechanism of CVQKDs over the seawater channel, and this research is expected to ensure unconditional security in underwater communications.

Additionally, in CVQKDs, there are two types of quantum state modulation: Gaussian modulation (GM) [14,15,16] and discrete modulation (DM) [17,18,19]. In continuous-variable quantum communication systems using discrete modulation, the phase shift keying (PSK) constellation is typically employed. However, research has shown that, in CVQKD systems, using such constellations cannot achieve the same communication distance and key rate as Gaussian modulation, thereby limiting the practical application capabilities of the communication system. In contrast, *N*-APSK modulation combines amplitude modulation and phase modulation, and, by increasing the number of modulatable quantum states, it can achieve communication performance close to that of Gaussian modulation. Almeida M et al. first proposed a communication scheme in 2021 that combines M symbol amplitude and phase shift keying with a CVQKD, demonstrating performance approaching that of Gaussian modulation in terms of transmission distance and key rate [20]. Building upon this foundation, Pereira D et al. proposed a novel 128-APSK constellation distribution structure. The experimental results demonstrated that this system can withstand collective attacks while transmitting information over distances exceeding 185 km [21]. As the research into *N*-APSK modulation technology in the field of CVQKDs continues to deepen, optimizing and adjusting different constellation diagrams and combining them with probabilistic constellation shaping technology can enhance the system’s anti-interference capabilities [22] and key rate [23], thereby effectively promoting the performance optimization of quantum communication systems in practical applications.

This paper designs a CVQKD proposal based on *N*-APSK modulation over the seawater channel. First, the characteristics and representation methods of the regular *N*-APSK constellation diagram are studied. Second, an optimal regular *N*-APSK constellation configuration scheme is designed based on the principle of maximizing the MED. Finally, the attenuation effects of quantum signals in the seawater channel, including absorption and scattering effects, are investigated. Based on this, simulations validate the performance of the *N*-APSK-modulated CVQKD scheme over the seawater channel. The simulation results show that the *N*-APSK-modulated CVQKD protocol achieves a longer transmission distance than the Gaussian modulation protocol over the seawater channel.

This paper is organized as follows: In Section 2, we describe the characteristics of the *N*-APSK constellation diagram and analyze *N*-APSK modulation based on the MED maximization principle while proposing a CVQKD based on *N*-APSK modulation over the seawater channel. In Section 3, we analyze the parameters of the seawater channel and simulate the transmission performance of the CVQKD scheme based on *N*-APSK modulation over the seawater channel. Finally, the conclusion is outlined in Section 4.

## 2. CVQKD Based on *N*-APSK Modulation

### 2.1. N-APSK Modulation

The *N*-APSK constellation consists of multiple concentric rings, with the same number of constellation points on each ring, and each ring is composed of *N*-APSK constellations. The main feature of the *N*-APSK constellation is that the number of constellation points on all rings is consistent and the phase changes of constellation points on different rings are consistent, with the entire constellation diagram showing a gradual expansion from the center outward. This consistency in amplitude and phase simplifies the modulation and measurement demodulation processes of quantum states in CVQKDs, thereby reducing the complexity of actual operations to a certain extent.

**Figure 1:**
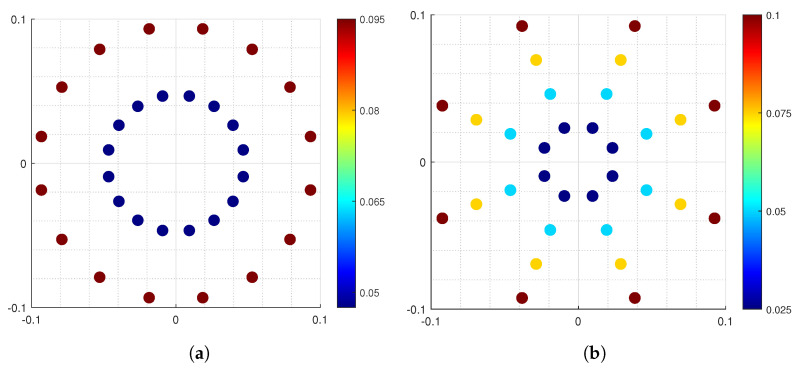
The diagram of 32–APSK constellation point distribution. (**a**) $\left(2^{4}\times 2^{1}\right)$ of 32–APSK. (**b**) $\left(2^{3}\times 2^{2}\right)$ of 32–APSK. The color bars represent the probability of each constellation point.

Based on the characteristics of the *N*-APSK constellation, define three non-negative integers *m*, $m_{1}$, and $m_{2}$, satisfying $m_{1}+m_{2}=m$ and $m_{1}\geq m_{2}$. Then, the number of constellation points in each ring is represented by $N_{R}=2^{m_{1}}$, and the number of rings is represented by $M=2^{m_{2}}$. Therefore, the total number of constellation points in the constellation diagram is $N=N_{R}\times M=2^{m_{1}}\times 2^{m_{2}}=2^{m}$. Thus, all *N*-APSKs can be represented by non-negative integers $m_{1}$ and $m_{2}$, denoted as $\left(2^{m_{1}}\times 2^{m_{2}}\right)$ of *N*-APSK. The two types of 32-APSK shown in Figure 1 can be represented as $\left(2^{4}\times 2^{1}\right)$ 32-APSK $\left(m_{1}=4,m_{2}=1\right)$ and $\left(2^{3}\times 2^{2}\right)$ 32-APSK $\left(m_{1}=3,m_{2}=2\right)$, respectively.

By utilizing the amplitude and phase information contained in the constellation points to represent their positions, the *N*-APSK constellation set $\chi_{N-APSK}$ can be formally expressed as

(1) $$\chi_{N-APSK}={r_{R}}^{j\left(\frac{2\pi }{N_{m}}-\frac{1}{2}\theta_{m}\right)},j=1,\ldots ,N_{m},$$

where $r_{R}$ represents the radius of the *R*-th ring, i.e., the distance between the constellation point and the center of the circle, which is determined by the modulation variance $V_{A}$ in subsequent performance analysis, and R=1,2,3,…,M. Since the number of constellation points in each ring is the same, it is denoted by $N_{m}$, where $N_{m}=2^{m_{1}}$. $\theta_{m}$ represents the phase shift of the constellation points in the ring, where $\theta_{m}=\frac{2\pi }{N_{m}}=\frac{2\pi }{2^{m_{1}}}$. During the modulation stage, the regular components of the quantum state are modulated, corresponding to the amplitude modulation and phase modulation of each constellation point, thereby establishing the association between the quantum state and the *N*-APSK modulation format.

Based on the above analysis, combined with the representation of quantum superposition states and the probability form of the Boltzmann–Maxwell distribution, the quantum state to be modulated can be represented as

(2) $${|\alpha_{1}\rangle }_{1}+\ldots +P_{1|N_{m}}\cdot {|\alpha_{N_{m}}\rangle }_{1}+\ldots +P_{M|1}\cdot {|\alpha_{1}\rangle }_{M}+\ldots +P_{M|N_{m}}\cdot {|\alpha_{N_{m}}\rangle }_{M},$$

where $P_{M|N_{m}}$ represents the probability of $N_{m}$ quantum states on the *M* ring, and all probabilities satisfy

(3) $${P_{1|1}}^{2}+\ldots +{P_{1|N_{m}}}^{2}+\ldots +{P_{M|1}}^{2}+\ldots +{P_{M|N_{m}}}^{2}=1,$$

As the number of constellation points *N* increases, the number of *N*-APSK constellation types also increases, making the selection of constellation types more complex. Additionally, under the same *N* value, differences in the number of rings and the distribution of constellation points within each ring result in variations in energy allocation and inter-constellation point distance intervals caused by the probability distribution of constellation points. These variations will impact the complexity of subsequent quantum state modulation and measurement demodulation, as well as channel capacity. Therefore, it is necessary to determine the optimal constellation form by maximizing the MED principle to optimize system performance [24]. MED is obtained by calculating the distances between constellation points within the same ring and between two constellation points in adjacent rings and then comparing them. First, for the distance $L_{R}$ between adjacent constellation points on the *R*-th ring, the square of the distance can be expressed as

(4) $$L_{R}^{2}=2r_{R}^{2}\left[1-\cos\left(\frac{2\pi }{N_{R}}\right)\right],$$

where $r_{R}=\sqrt{-\ln\left[1-2^{-m_{2}}\cdot \left(R-\frac{1}{2}\right)\right]}$. Secondly, the square of the distance between adjacent constellation points in adjacent rings *R* and *R* + 1 can be expressed as

(5) $$L_{R,R+1}^{2}=r_{R}^{2}+r_{R+1}^{2}-2r_{R}r_{R+1}cos\varphi ,$$

where φ represents the minimum phase shift between any pair of constellation points in ring *R* and ring *R* + 1, which can be expressed as

(6) $$\varphi =\min_{R,R+1}{\left|\theta_{R}-\theta_{R+1}\right|}^{2},$$

Finally, the MED of the *N*-APSK constellation is determined by minimizing Equations (5) and (6),

(7) $$L_{min}^{2}=min\left\{L_{R}^{2},L_{R,R+1}^{2}\right\},$$

Since the data results obtained when calculating the Euclidean distances between rings and between constellation points in different forms of *N*-APSK constellations may vary significantly, the harmonic mean of the Euclidean distances is used for the final result comparison to avoid the influence of individual extreme data on the overall results, thereby obtaining more balanced and stable results. The harmonic mean ${\bar{l}}_{ED}^{2}$ of the Euclidean distance can be expressed as [25]

(8) $$\frac{1}{{\bar{l}}_{ED}^{2}}=\frac{1}{m\cdot 2^{m}}\sum_{R=1}^{M}\left(\frac{1}{L_{R}^{2}}+\frac{1}{L_{R,R+1}^{2}}\right).$$

By calculating the harmonic mean ${\bar{l}}_{ED}^{2}$ of the MED for the 32-APSK constellation, the optimal rule *N*-APSK constellation is determined by maximizing ${\bar{l}}_{ED}^{2}$, as shown in Table 1. By comparison, it can be concluded that the $\left(2^{4}\times 2^{1}\right)$ of 32-APSK constellation map is better as it has the maximum harmonic mean.

### 2.2. The Proposal of CVQKD Based on *N*-APSK Modulation over Seawater Channel

**Figure 2:**
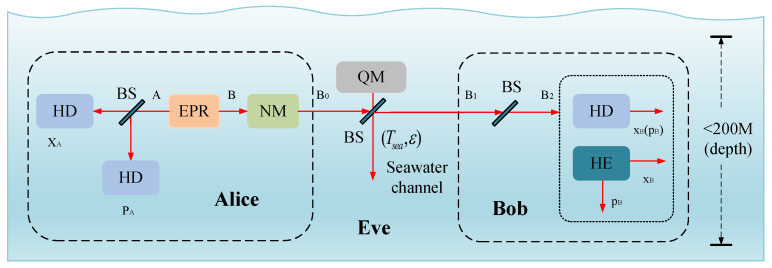
Seawater CVQKD based on *N*-APSK modulation model diagram. HD: homodyne detection; HE: heterodyne detection; EPR: Einstein–Podolsky–Rosen state; NM: *N*–APSK modulation; BS: beam splitter; $T_{sea}$, ε: channel parameter operator; QM: quantum memory.

This section will provide a detailed explanation of the seawater CVQKD scheme based on *N*-APSK modulation. Research has shown that *N*-APSK modulation is an efficient modulation scheme with high data rates and excellent interference resistance. By simultaneously adjusting the amplitude and phase of the signal, it can significantly enhance data carrying capacity. Additionally, since the seawater channel experiences greater attenuation compared to fiber optic or atmospheric channels, we will focus on analyzing the transmission performance of the *N*-APSK-modulated CVQKD proposal over the seawater channel in subsequent analyses. The CVQKD system based on *N*-APSK modulation is depicted in Figure 2. The prepare-and-measure (PM) scheme can be described as follows:

- Step 1: Alice generates a Gaussian random number sequence *S* based on the probability distribution of the *N*-APSK constellation points, then encodes the information to be transmitted onto the sequence *S*. Alice modulates the regular components $X_{A}$ and $P_{A}$ of the quantum state using the Gaussian random number sequence *S* to obtain the corresponding quantum state $|\alpha_{A}\rangle =|X_{A}+iP_{A}\rangle$, which is then sent to the seawater channel. Since the modulation of the regular position $X_{A}$ and regular momentum $P_{A}$ of the quantum state corresponds to the amplitude and phase modulation of the constellation points in *N*-APSK modulation, a correspondence is established between the quantum state and the Gaussian random number interval;
- Step 2: The optical properties (absorption and scattering) of the seawater channel cause significant attenuation of the transmitted quantum signal, with a transmittance of $T_{sea}$. Based on the above analysis, in short-distance transmission scenarios, this channel also introduces excess noise ε, which is mainly caused by environmental disturbances. Therefore, relative to the noise level at the channel input end, the total noise generated by the seawater channel can be expressed by the formula χch=11TseaTsea+ε−1;
- Step 3: At Bob’s side, an imperfect detector with efficiency η and electrical noise rate $v_{el}$ is used to measure the received quantum state. The noise introduced by the detector corresponding to Bob’s input end can be expressed as $\chi_{h}$ (in shot noise units) and satisfied as
  (9) $$\chi_{hom}=\left[\left(1-\eta \right)+v_{el}\right]/\eta ,$$
  for homodyne detection and
  (10) $$\chi_{het}=\left[\left(2-\eta \right)+2v_{el}\right]/\eta ,$$
  for hetrodyne detection. The total noise referred to the channel input can be expressed as $\chi_{\mathrm{tot}}=\chi_{ch}+\chi_{h}/T_{\mathrm{sea}}$.

## 3. Performance Analysis

### 3.1. Parameter Evaluation of Seawater Channel

The composition of seawater varies with a variety of factors, such as environment and region, making it difficult to study its propagation characteristics in detail using general models. On the one hand, the light intensity decreases sharply with the increase in the transmission distance of the quantum state in the seawater channel, so the light absorption effect is produced. On the other hand, the optical radiation area increases with the transmission distance of the quantum state, which in turn creates a light scattering effect. Absorption and scattering result in loss of light intensity and deflection of the beam, causing the energy of the optical signal received by the receiver to be continuously attenuated [26]. Therefore, this paper mainly considers the influence of light absorption and scattering effects on the transmittance of the system.

Depending on the composition of seawater, the absorption effect is mainly caused by the absorption of pure seawater, phytoplankton, colored dissolved organic matter, and non-algal particles (minerals and detritus) to light [27]. Now, using the chlorophyll-a based model, the spectral absorption coefficients are defined as

(11) $$\begin{array}{c} a\left(\lambda ,d\right)=a_{w}\left(\lambda \right)+a_{c}\left(\lambda \right)C_{c}{\left(d\right)}^{0.602}+a_{f}C_{f}\left(d\right)\exp\left(-k_{f}\lambda \right)\\ +a_{h}C_{h}\left(d\right)\exp\left(-k_{h}\lambda \right)+a_{d}\exp\left(-k_{d}\lambda \right), \end{array}$$

where λ and *d* represent wavelength and depth, respectively. $a_{w}\left(\lambda \right)$ and $a_{c}\left(\lambda \right)$ are the absorption coefficients of pure seawater and the spectral absorption coefficients of chlorophyll concentration, respectively, which can be derived from Ref. [28]. The spectral absorption coefficients and exponential coefficients of fulvic acid, humic acid, and non-algal particles are $a_{f}$ = 35.959 m^2^m2mgmg, $k_{f}$ = 0.0189 nm^−1^, $a_{h}$ = 18.828 m^2^m2mgmg, $k_{h}$ = 0.01105 nm^−1^, and $a_{d}$ = 9.721 m^2^m2mgmg, $k_{d}=0.012$ nm^−1^. $C_{f}$ and $C_{h}$ are the concentrations of fulvic acid and humic acid at depth *d* as follows

(12) $$\begin{array}{c} C_{f}\left(d\right)=1.74098C_{c}\left(d\right)\exp\left(0.12327C_{c}\left(d\right)\right),\\ C_{h}\left(d\right)=0.19334C_{c}\left(d\right)\exp\left(0.12343C_{c}\left(d\right)\right). \end{array}$$

$C_{c}\left(d\right)$ is the chlorophyll-a concentration at depth *d*, denoted as

(13) $$C_{c}\left(d\right)={\bar{Chla}}_{Zeu}\left(C_{b}-s\times \frac{d}{Z_{eu}}+C_{Max}\exp\frac{-{\left(d/Z_{eu}-\zeta_{Max}\right)}^{2}}{\Delta \zeta^{2}}\right),$$

where ${\bar{Chla}}_{Zeu}$ is the average column-integrated content of total chlorophyll-a within the euphotic layer, $C_{b}$ is the background chlorophyll-a concentration at the surface, *s* is the vertical gradient, $Z_{eu}$ is the depth of the euphotic layer, $C_{Max}$ is the maximum concentration, Δζ is the width of the Gaussian peak, and ζMax=CmaxCmaxZeuZeu is the dimensionless depth of the maximum concentration.

Similarly, the scattering effect of seawater on light is mainly caused by the scattering of pure seawater and non-algal particles. Therefore, the scattering coefficient can be expressed as

(14) $$b\left(\lambda ,d\right)=b_{w}\left(\lambda \right)+b_{s}\left(\lambda \right)C_{s}\left(d\right)+b_{l}\left(\lambda \right)C_{l}\left(d\right),$$

where the scattering spectra for pure water $b_{w}\left(\lambda \right)$, small particles $b_{s}\left(\lambda \right)$, and large particles $b_{l}\left(\lambda \right)$ are

(15) $$\begin{array}{c} b_{w}\left(\lambda \right)=0.005823{\left(400/\lambda \right)}^{4.3222},\\ b_{s}\left(\lambda \right)=1.1513{\left(400/\lambda \right)}^{1.7},\\ b_{l}\left(\lambda \right)=0.3411{\left(400/\lambda \right)}^{0.3}, \end{array}$$

$C_{s}\left(d\right)$ and $C_{l}\left(d\right)$ represent the concentrations of small and large particles at depth as follows

(16) $$\begin{array}{c} C_{s}\left(d\right)=0.01739C_{c}\left(d\right)\exp\left(0.11631C_{c}\left(d\right)\right),\\ C_{l}\left(d\right)=0.76284C_{c}\left(d\right)\exp\left(0.03092C_{c}\left(d\right)\right). \end{array}$$

**Figure 3:**
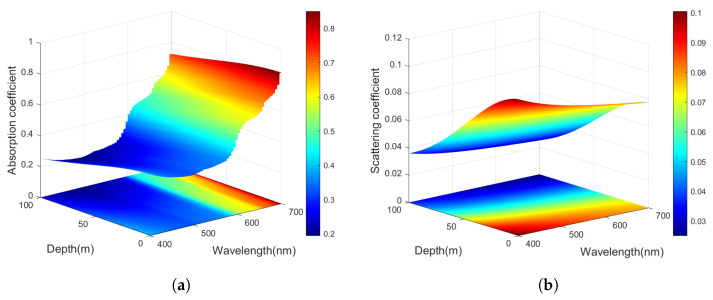
The functional relationship for absorption and scattering coefficients of the depth *d* and wavelength λ. (**a**) Absorption coefficient. (**b**) Scattering coefficient.

Figure 3 simulates the absorption and scattering coefficients of the seawater channel at different depths and wavelengths. The specific constant values are ${\bar{Chla}}_{Zeu}=0.250$, $Z_{eu}=80.2$, $C_{b}=0.570$, s=0.173, $C_{\max}=0.766$, and Δζ=0.814 [29]. From Figure 3a, it can be seen that the absorption coefficient decreases with the increase in seawater depth, and, with the increase in wavelength, the absorption coefficient increases first and then decreases. As shown in Figure 3b, the scattering coefficient is decreasing with increases in depth and wavelength.

The total seawater channel attenuation c(λ,d) can be derived as c(λ,d)=a(λ,d)+b(λ,d). From the above analysis, it can be seen that the propagation characteristics of seawater for light at different depths are different, and the change process is nonlinear. Therefore, for the sake of simplicity, we only consider the case that the signal light propagates on the horizontal surface at the same depth. Without loss of generality, we can assume that the propagation characteristics of light remain the same in the same horizontal plane. Therefore, the transmittance *T* is related to the total attenuation coefficient c(λ,d) and the transmission distance *L* as a function of the following, namely $T_{sea}=e^{-c(\lambda ,d)L}$.

In continuous-variable quantum communication systems, quantum noise characteristics are typically quantified using Gaussian-modulated noise units (SNUs). However, in marine channel transmission environments, the presence of complex background light noise interference within the water column may cause the actual measured excess noise at the receiver to exceed the initial noise level. Notably, background light noise in marine environments primarily originates from the interaction between solar radiation in the atmosphere (comprising direct sunlight and sky-scattered light) and seawater. This interaction induces phase mismatch in quantum light, thereby increasing the quantum bit error rate. Consequently, it is essential to investigate excess noise in seawater channels. Therefore, the solar background noise obtained from seawater can be expressed as

(17) $$P_{sea}=L_{sol}\times \Omega_{fov}\times \pi r_{0}^{2},$$

where $\Omega_{fov}=\pi$ and $r_{0}=1$ m are the field of view and radius of the the virtual telescope at the Bob side, respectively. $L_{sol}$ is the solar radiation of sunlight. The total excess noise is

(18) $$\varepsilon =\varepsilon_{0}+\tau \frac{P_{sea}}{h\nu },$$

where *h* is the Planck’s constant, τ=1 is the effective sampling period, and ν is the frequency of the noise photon.

### 3.2. Performance Analysis of CVQKD Based on *N*-APSK Modulation

In this section, we will analyze the security of the CV-QKD based on *N*-APSK modulation over the seawater channel under collective attacks in the asymptotic case. As discussed in Section 2.1, the *N*-APSK constellation is designed based on the MED maximization principle, with quantum states modulated to specific constellation positions. This design not only enhances the distinguishability between quantum state modulation and measurement demodulation, thereby improving the accuracy of measurement results, but also reduces the complexity of modulation and demodulation due to the consistent phase changes of constellation points in the *N*-APSK constellation, thereby enhancing the overall performance of the communication system. Therefore, we analyzed the performance of the CVQKD over the seawater channel using numerical simulations for two different 32-APSK modulation protocols and APSK modulation protocols with different *N* values.

Under asymptotic security analysis, the key rate of the protocol can be expressed as

(19) $$K\geq \beta I_{AB}-\chi_{BE},$$

where β is the negotiation efficiency, $I_{AB}$ is the Shannon mutual information between Alice and Bob, and $\chi_{BE}$ is the Holevo bound between the eavesdropper Eve and Bob. In the case of homodyne detection detection, $I_{AB}$ can be expressed as

(20) $$I_{AB}=\frac{1}{2}\log_{2}\left(\frac{V+\chi_{tot}}{1+\chi_{tot}}\right),$$

where $V=V_{A}+1$; the total noise $\chi_{tot}$ of the channel can be obtained from Section 2.2.

Before calculating the information $\chi_{BE}$ of Eve eavesdrops from Bob, we need to discuss the effect of parameter $Z_{BM}$ on the covariance matrix $\Gamma_{BM}$ between Alice and Bob under the Boltzmann–Maxwell distribution. When the Boltzmann–Maxwell distribution is selected, the density matrix $\tau_{BM}$ of quantum state ${|\alpha_{k_{R}}\rangle }_{R}$ can be expressed as a weighted mixture of quantum states:

(21) $$\tau_{BM}=\sum_{R=1}^{M}\sum_{k_{R}=1}^{N_{R}}P_{k_{R}|R_{BM}}{|\alpha_{k_{R}}\rangle }_{R}{\langle \alpha_{k_{R}}|}_{R},$$

where $P_{k_{R}|R_{BM}}$ represents the probability of selecting each quantum state. $Z_{BM}$ can be expressed as

(22) $$Z_{BM}=2\sqrt{T_{sea}}tr\left(\tau^{\frac{1}{2}}\alpha \tau^{\frac{1}{2}}\alpha^{†}\right)-\sqrt{2T_{sea}\chi_{tot}\omega },$$

and

(23) $$\omega =\sum_{R=1}^{M}\sum_{k_{R}=1}^{N_{R}}P_{k_{R}|R_{BM}}\left({\langle \alpha_{k_{R}}|}_{R}{\alpha_{\tau }}^{†}\alpha_{\tau }{|\alpha_{k_{R}}\rangle }_{R}-{\left|{\langle \alpha_{k_{R}}|}_{R}\alpha_{\tau }{|\alpha_{k_{R}}\rangle }_{R}\right|}^{2}\right),$$

where $\alpha_{\tau }=\tau^{\frac{1}{2}}\alpha \tau^{-\frac{1}{2}}$, $\tau =\tau_{BM}$, and α and $\alpha^{†}$ denote the annihilation operator and creation operator, respectively. Therefore, the covariance matrix $\Gamma_{BM}$ of the Boltzmann–Maxwell distribution can be expressed as

(24) $$\Gamma_{BM}=\left(\begin{array}{cc} aI_{2} & c\sigma_{z}\\ c\sigma_{z} & bI_{2} \end{array}\right)=\left(\begin{array}{cc} VI_{2} & Z_{BM}\sigma_{z}\\ Z_{BM}\sigma_{z} & T_{sea}\left(V+\chi_{tot}\right)I_{2} \end{array}\right),$$

where $\sigma_{z}$ represents the Pauli matrix and $I_{2}$ is a 2×2 unit matrix, $V_{A}$ is the modulation variance, and $\langle n\rangle$ represents the average number of photons.

In summary, considering a collective attack, the amount of information $\chi_{BE}$ that Eve eavesdrops from Alice can be expressed as

(25) $$\chi_{BE}=S\left(E\right)-S\left(\left.E\right|B\right),$$

where $S\left(E\right)$ is the von Neumann entropy of the system, and $S\left(\left.E\right|B\right)$ is the conditional entropy of the system. $S\left(E\right)$ can be obtained from the singular eigenvalues $\upsilon_{1}$ and $\upsilon_{2}$ of the covariance matrix $\Gamma_{BM}$:

(26) $$S\left(E\right)=G\left(\upsilon_{1}\right)-G\left(\upsilon_{2}\right),$$

where

(27) $$\begin{array}{c} \upsilon_{1,2}=\sqrt{\frac{1}{2}\left(A\pm \sqrt{A-4B^{2}}\right)},\\ A=V^{2}+{T_{sea}}^{2}{\left(V+\chi_{tot}\right)}^{2}-2T_{sea}{Z_{BM}}^{2},\\ B=T_{sea}V^{2}+T_{sea}V\chi_{tot}-T_{sea}{Z_{BM}}^{2}. \end{array}$$

$S\left(\left.E\right|B\right)$ can be obtained from the singular eigenvalues $\upsilon_{3}$ and $\upsilon_{4}$ of the covariance matrix $\Gamma_{BM}$:

(28) $$S\left(\left.E\right|B\right)=G\left(\upsilon_{3}\right)+G\left(\upsilon_{4}\right),$$

where

(29) $$\begin{array}{c} \upsilon_{3,4}=\sqrt{\frac{1}{2}\left(C\pm \sqrt{C^{2}-4D}\right)},\\ C=\frac{V+BT_{sea}\left(V+\chi_{tot}\right)+A}{V+1},\\ D=\frac{BT_{sea}\left(V+\chi_{tot}\right)+B^{2}}{V+1}. \end{array}$$

for $G\left(\upsilon_{i}\right)$, there existed

(30) $$G\left(\upsilon_{i}\right)=\left(\frac{\upsilon_{i}+1}{2}\right)\log_{2}\left(\frac{\upsilon_{i}+1}{2}\right)-\left(\frac{\upsilon_{i}-1}{2}\right)\log_{2}\left(\frac{\upsilon_{i}-1}{2}\right).$$

**Figure 4:**
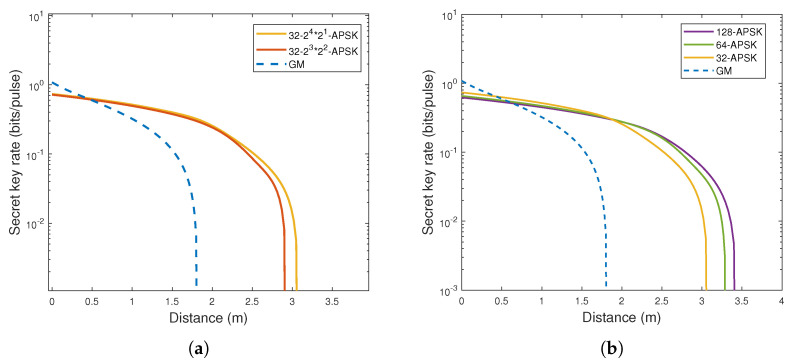
The functional relationship between transmission distance and key rate. (**a**) The functional diagram of different 32–APSK modulation schemes. (**b**) The functional diagram of different *N*–APSK modulation schemes.

By selecting appropriate modulation variance value $V_{A}$, this study investigates the relationship between communication distance and key rate in CVQKD protocols based on *N*-APSK modulation. In this paper, we choose the light with a wavelength of 520 nm to study the system performance of the CVQKD protocol. Figure 4a illustrates the relationship between transmission distance and key rate for two different 32-APSK modulation schemes. By comparing $\left(2^{4}\times 2^{1}\right)$ of 32-APSK, $\left(2^{3}\times 2^{2}\right)$ of 32-APSK, and the Gaussian modulation (GM) scheme, the advantages of the 32-APSK CVQKD protocol are analyzed. The research results indicate that, among the two 32-APSK modulation schemes, the $\left(2^{4}\times 2^{1}\right)$ of 32-APSK scheme achieves a higher key rate than other communication schemes and supports longer communication distances. This result validates the effectiveness of the 32-APSK constellation design based on the MED maximization principle.

Finally, we analyze CVQKD schemes using *N*-APSK modulation with different *N* values. Figure 4b illustrates the relationship between transmission distance and key rate for the CVQKD protocol based on *N*-APSK modulation when *N* is set to 32, 64, and 128, respectively. As shown in the figure, as *N* is successively set to 32, 64, and 128, the key rate of the *N*-APSK CVQKD scheme gradually exceeds that of the Gaussian modulation scheme, and, as N increases, the communication distance further extends.

## 4. Conclusions

We propose a CVQKD based on *N*-APSK modulation over the seawater channel. In the *N*-APSK modulation scheme, we design the optimal *N*-APSK modulation scheme based on the MED maximization principle. At the same time, we analyze the attenuation effects of quantum signals in the seawater channel, including absorption effects and scattering effects. Based on this, simulations verify the performance of the CVQKD scheme based on *N*-APSK modulation over the seawater channel. The simulation results confirm that the *N*-APSK modulation scheme designed based on the MED maximization principle achieves a longer communication distance compared to schemes without this principle. Furthermore, as the *N* value increases, the communication distance of the CVQKD based on *N*-APSK modulation over the seawater channel gradually increases. In subsequent research on the CVQKD based on *N*-APSK modulation, the probability shaping scheme for *N*-APSK constellation points can be further optimized. By leveraging optimization algorithms such as dynamic programming, genetic algorithms, and matched entropy methods, the probability distribution of the signal can be adjusted to match the characteristics of the channel. This approach enhances transmission efficiency and maximizes channel capacity. 

## Figures and Tables

**Table 1:** The MED and the harmonic mean ${\bar{l}}_{ED}^{2}$ for 32–APSK.

32-APSK	MED	${\bar{l}}_{\mathrm{ED}}^{2}$
$\left(2^{4}\times 2^{1}\right)$	0.0438	1.0666
$\left(2^{3}\times 2^{2}\right)$	0.0782	0.6846

## Data Availability

All data generated or analyzed during this study are included in this published article.
